# Supplementary figures and images for: Interferon-inducible guanylate-binding protein 5 inhibits replication of multiple viruses by binding to the oligosaccharyltransferase complex and inhibiting glycoprotein maturation
Source: mBio. 2025 Nov 17;16(12):e02930-25. doi: 10.1128/mbio.02930-25 (PMC12691697; doi:10.1128/mbio.02930-25)

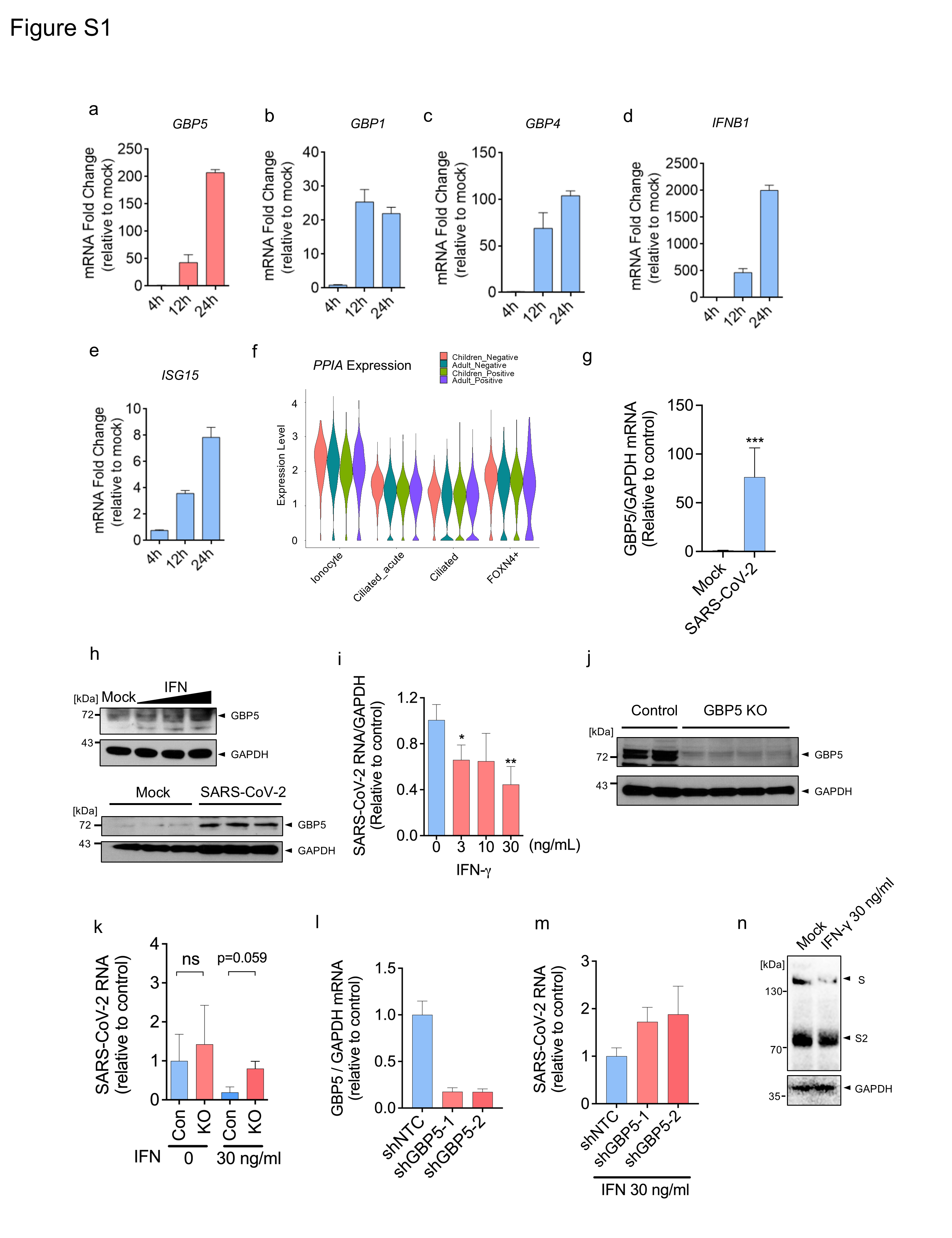

Supplement: Figure S1 — GBP5 is induced by SARS-CoV-2 infection of lung epithelial cells. [file mbio.02930-25-s0001.tif]

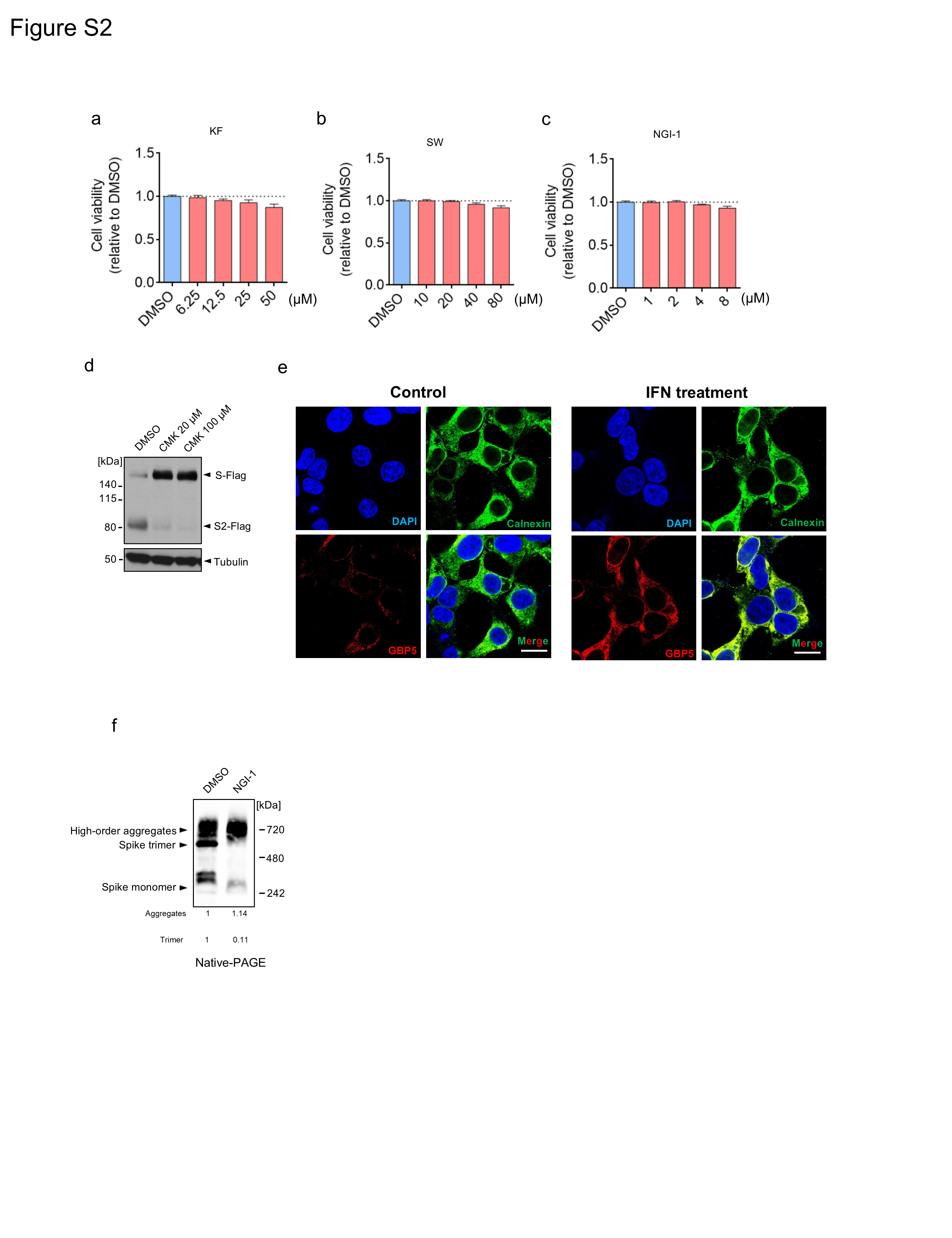

Supplement: Figure S2 — Cytotoxicity evaluation of glycosylation inhibitors, inhibition of SARS-CoV-2 S protein cleavage by CMK, and NGI-1-induced misfolding of S protein. [file mbio.02930-25-s0002.tif]

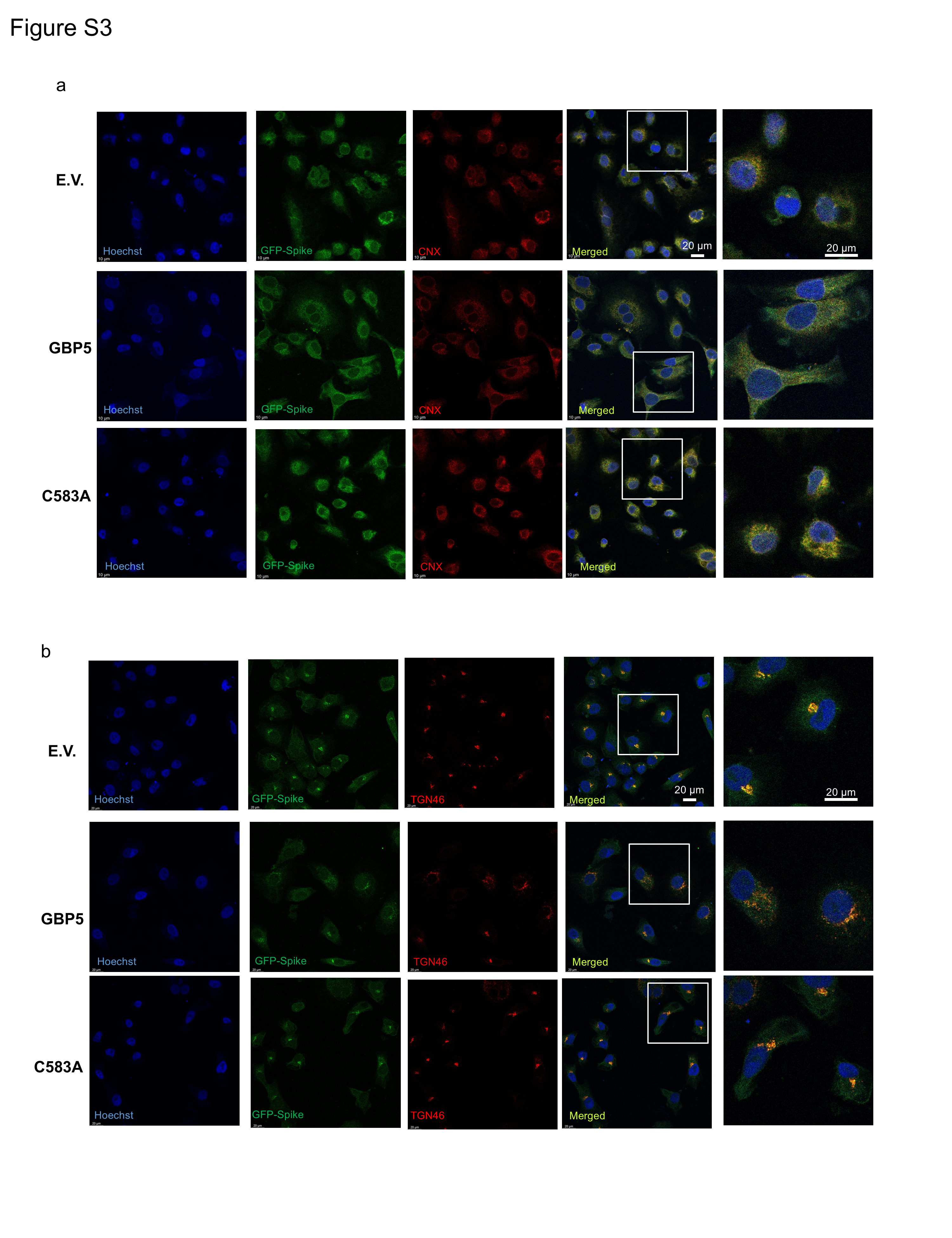

Supplement: Figure S3 — GBP5 overexpression inhibits ER-to-Golgi trafficking of the spike protein in A549-ACE2 cells. [file mbio.02930-25-s0003.tif]

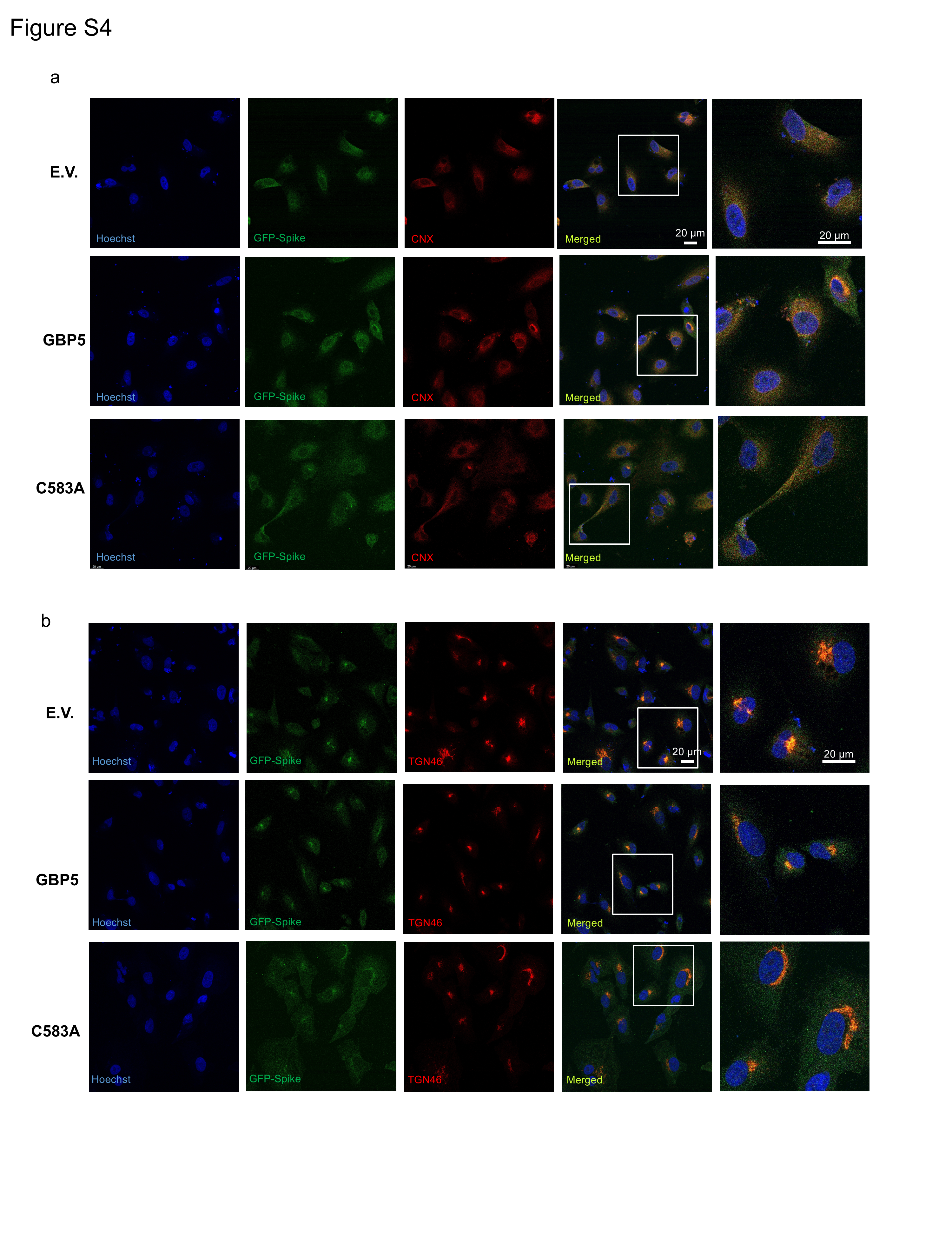

Supplement: Figure S4 — GBP5 overexpression inhibits ER-to-Golgi trafficking of the spike protein in 293T cells. [file mbio.02930-25-s0004.tif]

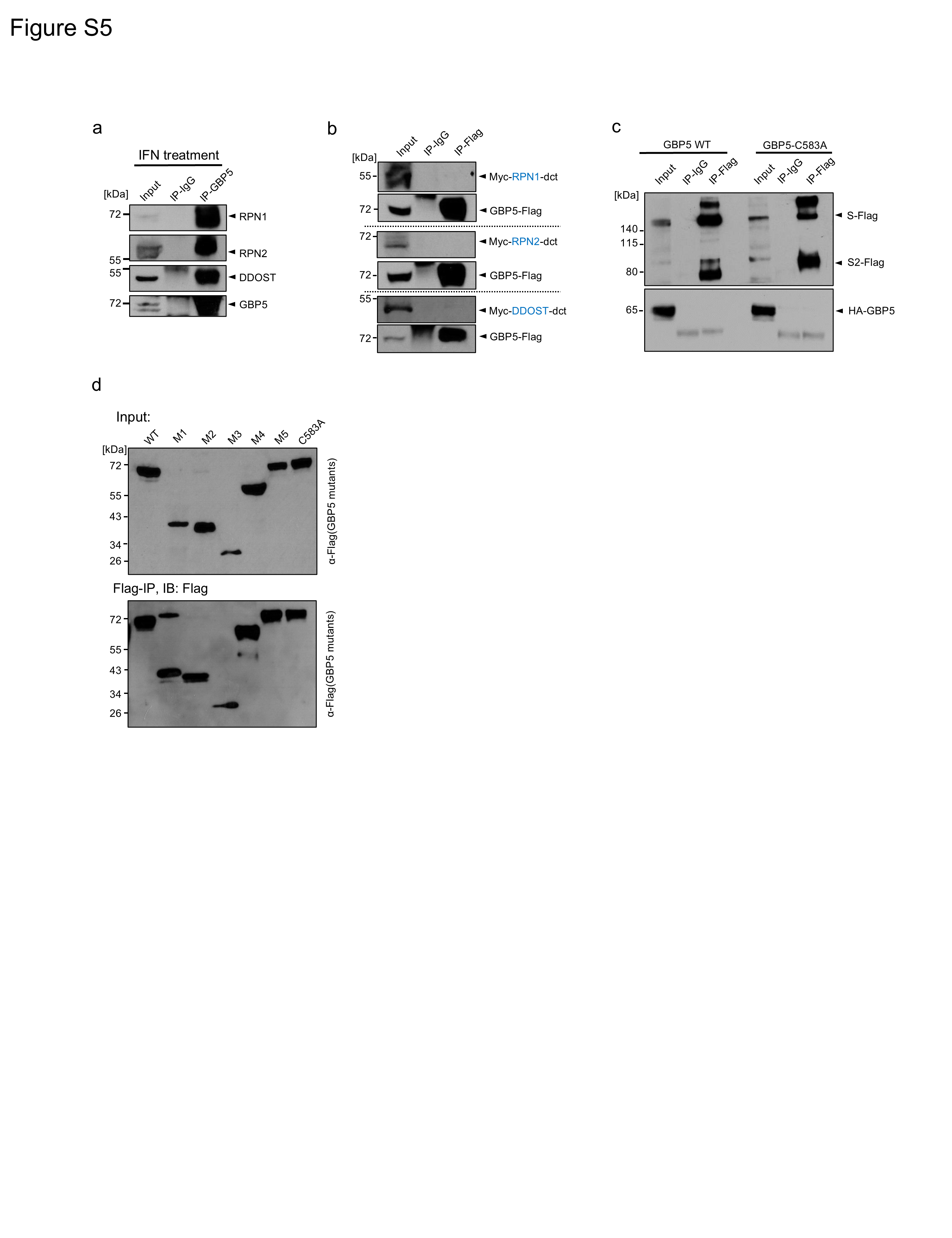

Supplement: Figure S5 — GBP5 does not interact with SARS-CoV-2 S protein. [file mbio.02930-25-s0005.tif]

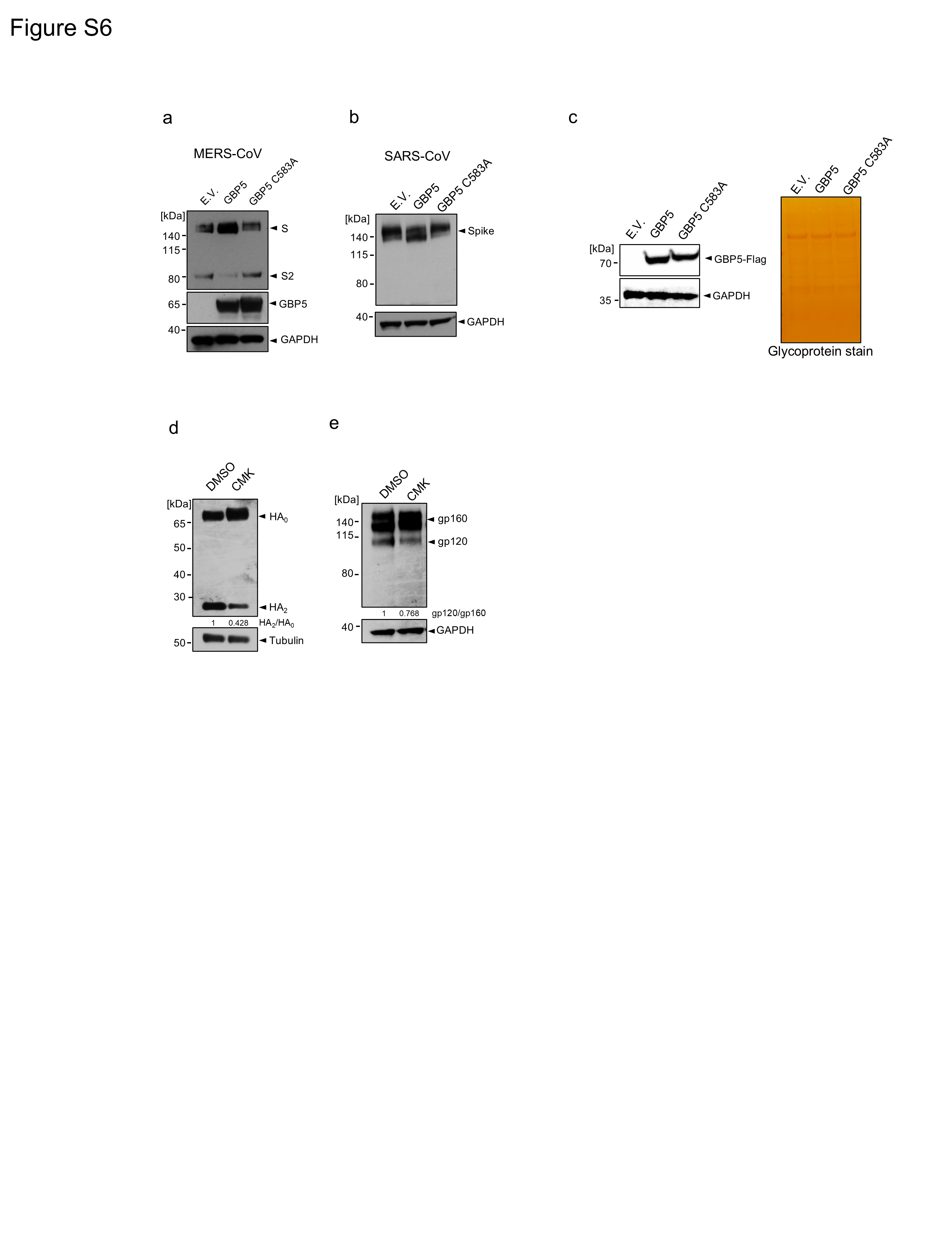

Supplement: Figure S6 — GBP5 suppresses glycosylation and cleavage of MERS-CoV and SARS-CoV S proteins and CMK inhibits furin-mediated S protein cleavage. [file mbio.02930-25-s0006.tif]

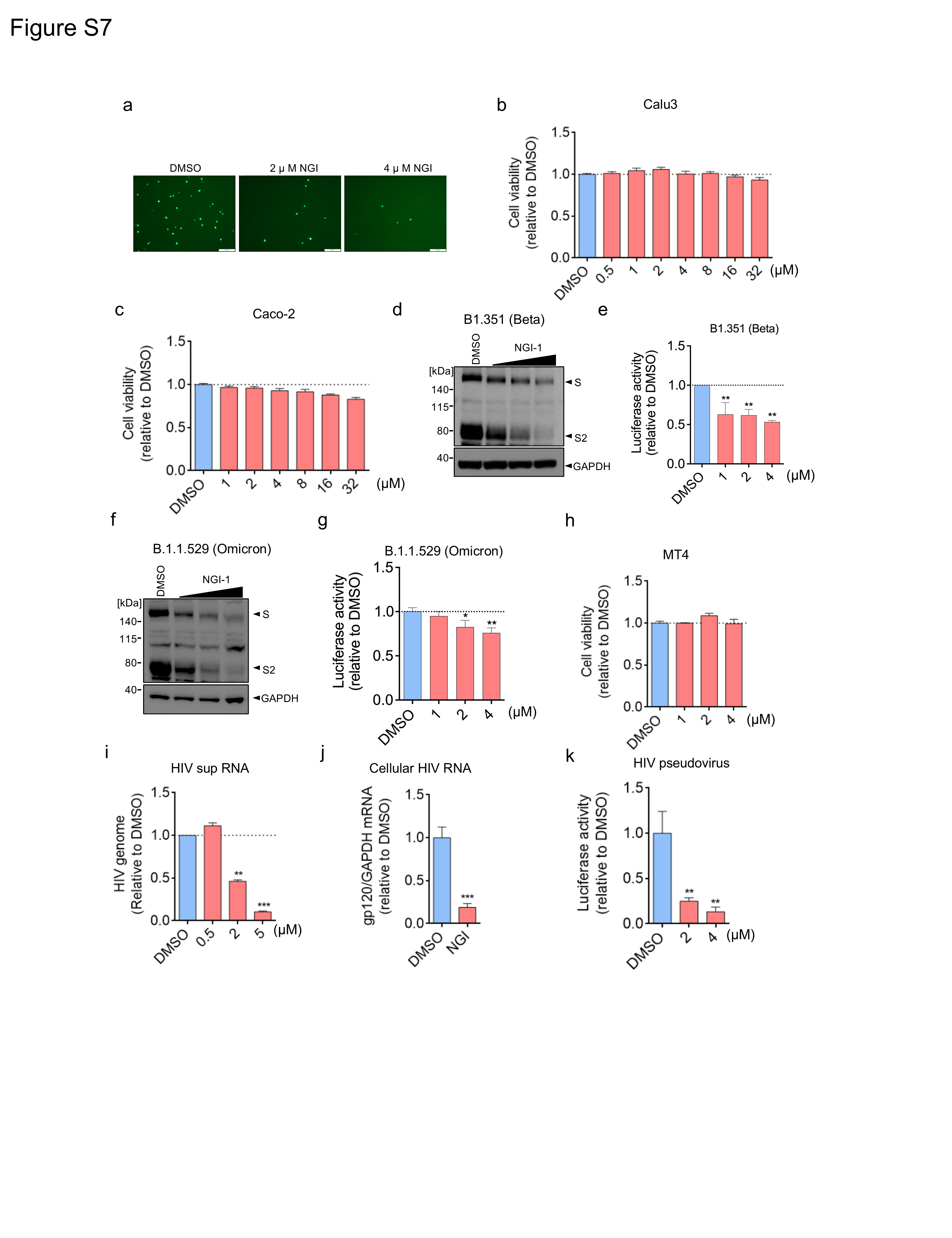

Supplement: Figure S7 — Host cell cytotoxicity and antiviral activity of NGI-1. [file mbio.02930-25-s0007.tif]
