# Supplementary material for: Interferon-inducible guanylate-binding protein 5 inhibits replication of multiple viruses by binding to the oligosaccharyltransferase complex and inhibiting glycoprotein maturation
Source: mBio. 2025 Nov 17;16(12):e02930-25. doi: 10.1128/mbio.02930-25 (PMC12691697; doi:10.1128/mbio.02930-25)
Supplement: Legends — Supplemental figure legends. [file mbio.02930-25-s0008.docx]

**SUPPLEMENTARY FIGURE LEGENDS**

**Figure S1. GBP5 is induced by SARS-CoV-2 infection of lung epithelial cells.**

a–e. RT-qPCR analysis of GBP5 (a), GBP1 (b), GBP4 (c), IFNB1 (d), and ISG15 (e) mRNA levels in Calu-3 cells infected for 4, 12, or 24 h with SARS-CoV-2 WT at an MOI of 0.33. Mean ± SD of n = 2. Fold change in expression is relative to mock-infected cells. Data are from reference ^63^.

f. Expression of the internal control gene PPIA in nasal samples from healthy or SARS-CoV-2 infected children and adults (see Figure 1 for details).

g-h RT-qCPR (g) and western blot analysis (h) of GBP5 induction by SARS-CoV-2 infection or IFN-γ treatment.

i. Calu3 cells were pretreated with different concentrations of IFN-γ for 16 h and infected with SARS-CoV-2 USA-WA1/2020 strain at a MOI of 0.1. After 48h, cells were collected and viral RNA in cell extracts was quantified by RT-qPCR. Mean ± SD of n = 3. *p < 0.05, **p < 0.01 by Student’s t-test.

j. GBP5 KO or control Calu3 cells were treated with IFN-γ for 16 h, and the knockout efficiency of GBP5 was validated by western blot analysis.

k. The viral RNA in the supernatant (see Figure 1 m-n for details) was quantified by RT-qPCR. Mean ± SD of n = 3.

l-m GBP5 or control shRNA transduced human primary bronchial epithelial cells (NHBE) were infected with USA-WA1/2020 strain at a MOI of 0.1. The knockdown efficiency of GBP5 was validated by qPCR (l). The viral RNA in the supernatant was quantified by RT-qPCR (m). Mean ± SD of n = 3.

n. 293T cells were transfected for 24 h with SARS-CoV-2 S plasmid and then treated with IFN-γ for 16 h. Blots were probed with an antibody that recognizes the cleaved S2 subunit and full-length S protein. GAPDH was probed as a loading control.

**Figure S2. Cytotoxicity evaluation of glycosylation inhibitors, inhibition of SARS-CoV-2 S protein cleavage by the furin inhibitor CMK, and glycosylation inhibitor NGI-1 induces misfolding of S protein.**

a–c. CellTiter-Glo 2.0 cell viability assay of 293T cells incubated for 24 h with DMSO or the indicated concentrations of the glycosylation inhibitors kifunensine (a), swainsonine (b), and NGI-1 (c). Mean ± SD of n = 2.

d. Western blot analysis of 293T cells transfected with SARS-CoV-2 S plasmid and incubated with DMSO or CMK for 48 h. Lysates were immunoprecipitated with anti-FLAG antibody and blots were probed with antibodies against FLAG or tubulin (loading control).

e. IFN-induced endogenous GBP5 is present in the ER. Fluorescence microscopy of 293T cells treated with or without IFN-γ and stained with antibodies against the ER marker calnexin and GBP5. Nuclei were stained with Hoechst 33342. Scale bar, 50 μm.

f. NGI-1 induces misfolding of S protein. 293T cells were transfected for 24 h with SARS-CoV-2 S and then incubated for 6 h with DMSO and 2 μM NGI-1 and subjected to native PAGE. Blots were probed with anti-S protein antibody.

**Figure S3. GBP5 overexpression inhibits ER-to-Golgi trafficking of the spike protein in A549-ACE2 cells**

a. GBP5 overexpression induces the retention of S protein in the ER. A549-ACE2 cells were transfected for 24 h with GBP5 or GBP5-C583A and then infected with USA-WA1/2020 strain at a MOI of 0.1 for 24 h. Fluorescence microscopy of cells stained with antibodies against the ER marker calnexin and Spike. Nuclei were stained with Hoechst 33342. Scale bar, 20 μm.

b. GBP5 overexpression reduces the distribution of S protein in the Golgi apparatus. A549-ACE2 cells were transfected for 24 h with GBP5 or GBP5-C583A and then infected with USA-WA1/2020 strain at a MOI of 0.1 for 24 h. Fluorescence microscopy of cells stained with antibodies against the Golgi marker TGN46 and Spike. Nuclei were stained with Hoechst 33342. Scale bar, 20 μm.

**Figure S4. GBP5 overexpression inhibits ER-to-Golgi trafficking of the spike protein in 293T cells**

a. GBP5 overexpression induces the retention of S protein in the ER. 293T cells were co-transfected with GBP5 or GBP5-C583A and FLAG-SARS-CoV-2 S plasmids for 48 h. Fluorescence microscopy of cells stained with antibodies against the ER marker calnexin and Spike. Nuclei were stained with Hoechst 33342. Scale bar, 20 μm.

b. GBP5 overexpression reduces the distribution of S protein in the Golgi apparatus. 293T cells were co-transfected with GBP5 or GBP5-C583A and FLAG-SARS-CoV-2 S plasmids for 48 h. Fluorescence microscopy of cells stained with antibodies against the Golgi marker TGN46 and Flag. Nuclei were stained with Hoechst 33342. Scale bar, 20 μm.

**Figure S5. GBP5 does not interact with SARS-CoV-2 S protein.**

a. IFN-induced endogenous GBP5 interacts with endogenous accessory subunits. 293T cells were stimulated with 100 ng/ml IFN-γ and cell lysates were immunoprecipitated with anti-GBP5 antibody and western blots were probed with antibodies against RPN1, RPN2, DDOST or GBP5.

b. GBP5 interacts with the OST complex from the cytoplasmic side. FLAG-tagged GBP5 and MYC-tagged RPN1, RPN2 or DDOST with removed C-terminal cytoplasmic regions were co-transfected into 293T cells, respectively. The cell lysates were immunoprecipitated with anti-FLAG antibody and western blots were probed with antibodies against MYC tag.

c. Western blot analysis of 293T cells transfected for 48 h with FLAG-SARS-CoV-2 S protein and either HA-GBP5 or HA-GBP5-C583A. Cell lysates were immunoprecipitated with anti-FLAG antibody and blots were probed with antibodies against HA or S protein.

d. The input and FLAG-IP efficiency of WT GBP5 and GBP5 mutants were shown (see Figure 3i for details)

**Figure S6. GBP5 suppresses glycosylation and cleavage of MERS-CoV and** **SARS-CoV S proteins and CMK inhibits furin-mediated S protein cleavage**.

a, b. Western blot analysis of 293T cells transfected for 48 h with empty vector (EV), GBP5. or GBP5-C583A together with either MERS-CoV (a) or SARS-CoV (b) S proteins. Blots were probed with an antibody that recognizes the S2 subunit and full-length S protein or with anti-GAPDH (loading control).

c. Western blot and glycoprotein stain analysis of 293T cells transfected for 48 h with empty vector (EV), GBP5. or GBP5-C583A. Blots were probed with were probed with antibodies against FLAG or with anti-GAPDH (loading control). Glycosylated proteins were stained in polyacrylamide gels using the periodic acid-Schiff (PAS) method.

d, e. Western blot analysis of 293T cells transfected with FLAG-IAV HA or with HIV-1 gp160 and incubated with 100 μM CMK or DMSO for 24 h (HA) or 48 h (gp160). Blots were probed with antibodies against FLAG (c), gp120 (d), and either tubulin or GAPDH (loading controls).

**Figure S7. Host cell cytotoxicity and antiviral activity of NGI-1.**

a. EGFP-expressing VSV pseudovirus was produced in 293T cells transfected with SARS-CoV-2 S protein and then treated with DMSO or NGI-1. After 24 h, the supernatants were collected and added to Calu-3 cells for 2 h. Infectivity of Calu-3 cells was assessed by fluorescence microscopy. Scale bar, 100 μm.

b, c. Cell viability assays of Calu-3 (B) and Caco-2 (C) cells treated for 48 h with DMSO or the indicated concentrations of NGI-1. Mean ± SD of n = 2.

d–g. Luciferase-expressing VSV pseudoviruses were produced in 293T cells transfected with B.1.351 (Beta) (d, e) or B.1.1.529 (Omicron) (f, g) S proteins and then treated with DMSO or 1, 2, or 4 μM NGI-1. After 24 h, the cells and supernatants were collected. Cell lysates were analyzed by western blotting with anti-S protein (d, f). Cell supernatants were added to Calu-3 cells for 2 h, and infectivity was assessed by measuring luciferase activity (e, g). Mean ± SD of n = 3. **p < 0.01, ***p < 0.001 by Student's t-test.

h. Cell viability assay of MT4 cells treated with DMSO or the indicated concentrations of NGI-1 for 48 h. Mean ± SD of n = 2.

i, j. MT4 cells were infected with HIV-1 LAI at an MOI of 0.01 and treated with DMSO or the indicated concentrations of NGI-1 for 48 h. HIV RNA in the supernatant (i) and the ratio of HIV gp120 RNA to cellular GAPDH mRNA (j) were analyzed by RT-qPCR. Mean ± SD of n = 3. **p < 0.01, ***p < 0.001 by Student's t-test.

k. Luciferase-expressing VSV pseudovirus was produced in 293T cells transfected with HIV gp160 and pNL4-3.Luc.R-E and then treated with DMSO or NGI-1 for 48 h. Cell supernatants were collected and added to MT4 cells for 1 h. Infectivity was assessed by measuring luciferase activity. Mean ± SD of n = 3. **p < 0.01 by Student's t-test.
